# Atomistic theory of thermally activated magnetization processes in Nd_2_Fe_14_B permanent magnet

**DOI:** 10.1080/14686996.2021.1942197

**Published:** 2021-09-06

**Authors:** Seiji Miyashita, Masamichi Nishino, Yuta Toga, Taichi Hinokihara, Ismail Enes Uysal, Takashi Miyake, Hisazumi Akai, Satoshi Hirosawa, Akimasa Sakuma

**Affiliations:** a The Institute for Solid State Physics (ISSP), The University of Tokyo, Kashiwa, Japan; b Research Center for Advanced Measurement and Characterization, National Institute for Materials Science (NIMS), Tsukuba, Japan; c Elements Strategy Initiative Center for Magnetic Materials, Research Center for Magnetic and Spintronics Materials, National Institute for Materials Science (NIMS), Tsukuba, Japan; d The National Institute of Advanced Industrial Science and Technology (AIST), Tsukuba, Japan; e Department of Applied Physics, Tohoku University, Sendai, Japan

**Keywords:** Coercivity, thermal fluctuation, finite temperature, stochastic LLG equation, Monte Carlo method, dipole–dipole interaction, 40 Optical, magnetic and electronic device materials; 203 Magnetics / Spintronics / Superconductors; 400 Modeling/Simulations

## Abstract

To study the temperature dependence of magnetic properties of permanent magnets, methods of treating the thermal fluctuation causing the thermal activation phenomena must be established. To study finite-temperature properties quantitatively, we need atomistic energy information to calculate the canonical distribution. In the present review, we report our recent studies on the thermal properties of the Nd_2_Fe_14_B magnet and the methods of studying them. We first propose an atomistic Hamiltonian and show various thermodynamic properties, for example, the temperature dependences of the magnetization showing a spin reorientation transition, the magnetic anisotropy energy, the domain wall profiles, the anisotropy of the exchange stiffness constant, and the spectrum of ferromagnetic resonance. The effects of the dipole–dipole interaction (DDI) in large grains are also presented. In addition to these equilibrium properties, the temperature dependence of the coercivity of a single grain was studied using the stochastic Landau-Lifshitz-Gilbert equation and also by the analysis of the free energy landscape, which was obtained by Monte Carlo simulation. The upper limit of coercivity at room temperature was found to be about 3 T at room temperature. The coercivity of a polycrystalline magnet, that is, an ensemble of interactinve grains, is expected to be reduced further by the effects of the grain boundary phase, which is also studied. Surface nucleation is a key ingredient in the domain wall depinning process. Finally, we study the effect of DDI among grains and also discuss the distribution of properties of grains from the viewpoint of first-order reversal curve.

## Graphical abstract

**Figure:**
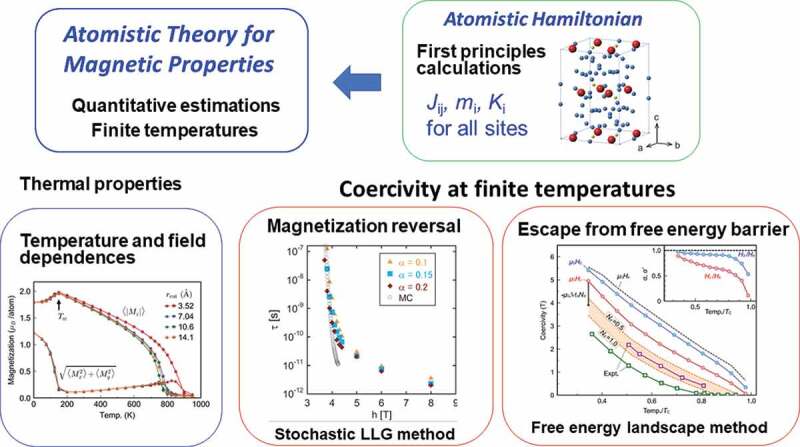

## 1. Introduction

The neodymium (Nd) polycrystalline magnet consisting of Nd_2_Fe_14_B [1–14] is an important high-performance permanent magnet. Because of its high coercivity, it is widely used for electric motors, electronic devices, and so forth [15]. Coercivity at finite temperatures is a key factor affecting the performance of permanent magnets. Coercivity essentially depends on the structure of grains and grain boundaries, and the nucleation of reversed magnetization and the depinning mechanisms of magnetic domain walls in the structure play an important role in coercivity [15–17]. Trials toward achieving higher coercivities at higher temperatures have been actively performed [18,19], but the quantitative properties of coercivity at finite temperatures have not been well understood [16].

In previous works, temperature effects have been taken into account by using the temperature-renormalized parameters, for example the exchange stiffness constant A(t) and the magnetic anisotropy energy K(T), which are obtained experimentally or by mean-field analyses. Coercivity at finite temperatures is, however, a phenomenon involving the breakdown of a metastable magnetic state. Magnetization reversal occurs with thermal agitation and thus it is a stochastic process [20]. To study such effects quantitatively, we need to take into account the effect of entropy. For this purpose, we must treat the temperature precisely. In thermal equilibrium state, the probability of a state i, $P_{eq}(i)$, is given by the canonical ensemble with the Boltzmann factor:

(1) $$P_{eq}(i)=\frac{1}{Z}e^{-\beta H(i)},\;Z=\sum_{i:allstates}e^{-\beta H(i)},\beta =\frac{1}{k_{B}T},$$

where H is the Hamiltonian representing the system.

If we use a coarse-grained Hamiltonian of the system, such as the continuum model, the definition of the degrees of freedom of the state is ambiguous and the above-mentioned probability is difficult to apply. Namely, if we study finite-temperature properties by micromagnetic simulation with continuous spin having temperature-dependent parameters, such as A(T) and K(T), the additional noise causes the double counting of the effect of thermal agitation. To appropriately take into account the temperature effect, we must use standard statistical mechanical methods with the canonical ensemble. To obtain the Boltzmann factor, one needs an atomistic Hamiltonian, which will be explained in Section 2. Using the atomistic Hamiltonian, the thermal effect is taken into account in the Monte Carlo (MC) method by the detailed balance condition and in the SLLG method by the fluctuation dissipation relation, which guarantees realization of the thermal equilibrium state at given temperatures as explained in Refs. [21,22].

Thus, it is necessary to use an atomistic model, and its Hamiltonian must be kept unchanged with the temperature. One may consider cases in which the lattice of the system changes with the temperature (expansion or shrinkage), which causes changes in parameters in the Hamiltonian. In such cases, we must introduce some additional compromised treatments. However, in the present work, we concentrate on the cases where we can ignore such an effect.

**Figure 1.**
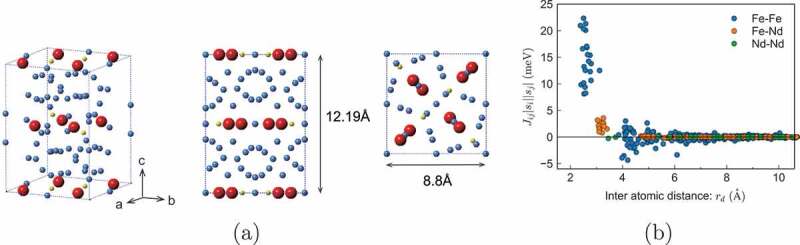
(a) (left) Unit cell of Nd_2_Fe${\;}_{14}$B. Neodymium, iron, and boron atoms are denoted by red, blue, and yellow spheres, respectively. The lattice constants [2] for the a-, b-, and c-axes are $d_{a}=d_{b}=8.80$ Å, and $d_{c}=12.19$ Å, respectively. (middle) Side view (from the a – or b-axis). (right) Top view (from the c-axis). (b) Exchange coupling constants between the atoms as a function of the distance

In the present paper, we review our recent works on finite-temperature properties. As mentioned above, an atomistic Hamiltonian for a material is necessary to study its thermal properties. Thus, we constructed an explicit atomistic Hamiltonian. Nd${\;}_{2}$Fe${\;}_{14}$B which is the main phase in the neodymium magnet, has a complex structure. The unit cell contains nine different sites with 68 atoms as depicted in Figure 1(a). Although, in general, the determination of the atomistic Hamiltonian is difficult even with the most sophisticated first-principles calculations, we constructed an atomistic Hamiltonian using the latest knowledge of microscopic parameters to reproduce the known thermodynamic properties as explained in Section 2.

Using the Hamiltonian, we first studied various thermal properties of the Nd_2_Fe${\;}_{14}$B magnet, such as magnetization. As an important characteristic of the magnet, we also studied the temperature dependence of anisotropy energy, as well as the temperature dependence of the threshold field of the magnetization reversal (coercivity) from the temperature dependence of the free energy as a function of the direction of magnetization by constrained MC simulation [23]. The dependence of the exchange stiffness constant on the direction reflecting the anisotropy of the crystal was also studied [24].

In addition to the above-mentioned macroscopic quantities, it has been found that the model can produce the microscopic magnetic structures of the magnet, for example, domain wall profiles, and also their temperature dependence [25]. These results well reproduce the experimental data, and thus we confirmed the validity of the model. Similar works with an atomistic model for the finite-temperature properties of magnetization and the domain wall have been recently reported by Gong and coauthors [26–28], who obtained thermodynamic quantities and also an effective continuous model. Using the model, they obtained the dependence of stiffness constants on the direction and the coercivity of a system with a grain boundary phase as mentioned above [24].

Furthermore, the atomistic model can produce the spectrum of ferromagnetic resonance(FMR) by stochastic Landau-Lifshitz-Gilbert (SLLG) simulation [22,29]. The effects of the dipole–dipole interaction (DDI) at room temperature were also studied [30].

Hard-magnetic compounds consist of many grains, and the reversal of magnetization under a reverse magnetic field close to the coercivity occurs as a cascade of reversals of magnetizations of grains. The process has been categorized into two processes. That is, the nucleation of reversal magnetization and the propagation of reversal magnetization across the grain boundary (referred to as the depinning of the domain wall). The overall process of the reversal is very complex, and cannot be studied by atomistic simulation.

Thus, we first study the reversal phenomena in atomistic models in a nanoscale system. These reversal phenomena are governed by the emergence of nuclei of nanometer-order magnetic domains (activation volume [31]) according to the classical Arrhenius-type analysis of the time dependence of magnetization. The single-grain coercivity thus obtained gives a theoretical upper limit for the coercivity at a given temperature, although it should be reduced further by the effect of interactions between grains in bulk magnets. It is widely accepted that coercivity is not an intrinsic property of hard magnets and that nucleation occurs at a defect. The reversal of magnetization propagates to hard-magnet grains, for which the pinning of the domain wall at the boundary phase is important. In this depinning process, the propagation of reversal is also governed by the surface nucleation of each grain of a hard magnet, for which the information from nanosize reversal is important.

Note that in contrast to the above-mentioned thermodynamic quantities, which we can calculate using (1), we do not have any explicit theoretical formula to calculate coercivity. Coercivity is the threshold of the magnetic field of the metastable magnetic state in the magnetic field in the opposite direction. At T=0 without thermal fluctuation, this threshold is uniquely defined. However, at finite temperatures, relaxation occurs stochastically through nucleation. There, the relaxation time is widely distributed. In this manner, coercivity is a highly non-equilibrium property, which prevents us from calculating it theoretically. Coercivity is phenomenologically defined as the magnetic field at which the relaxation time of magnetization is 1s [32–37]. We have approached this problem by the following methods.

(1) We studied the dynamics of relaxation by the SLLG method, which incorporates thermal fluctuations [22]. In this approach, the following difficulties exist, which are general problems in microscopic molecular simulations. First, the relaxation depends on the damping constant α of the LLG equation, which is difficult to know precisely. Moreover, the maximum relaxation time that can be obtained by atomistic calculation is limited. Indeed, the time scale of spin precession in a field of 1 T is of the $10^{-12}$s order, and the maximum time of simulation is up to several nanoseconds. These difficulties have prevented us from estimating the relaxation time quantitatively. However, we have been able to overcome these difficulties and obtained a quantitative estimation of the threshold field for a relaxation time of 1s [38].

(2) Alternatively, we approached relaxation phenomena from the viewpoint of the free energy barrier at finite temperatures. There have been some works focusing on the free energy barrier using the minimum energy path method [39], which enabled the energy along a path of evolution of magnetization from the metastable state to the stable state to be obtained, where the energy function contains temperature-dependent parameters. For this method, an explicit form of the free energy as a function of the configuration is necessary. Thus far, energy functionals of magnetization with temperature-dependent parameters have been used for this purpose, which may not appropriately express thermal fluctuations. On the other hand, we have developed a method of studying the metastable situation quantitatively at finite temperatures [40], for which we obtained the free energy as a function of magnetization from the atomistic Hamiltonian at a given temperature by using of the Wang–Landau method [41]. Using the explicit form of F(M), we estimated coercivity with and without the activation effect, and also clarified the concept of the activation volume introduced in the literature [32–34,42].

With these approaches, we have estimated coercivity at room temperature around 3 T for a single grain with the shape of a 10∼20 nm cube. The estimated threshold (∼ 3 T) gives the theoretical upper limit, which is very low compared with the zero-temperature coercivity and also smaller than the theoretical estimate of 2K(T)/M(T) for the uniform rotation of magnetization without thermal activation. Thus, the thermal fluctuation has been found to be one of the important ingredients contributing to the Kronmüller’s discrepancy [5], although the existence of magnetic inhomogeneity with reduced magnetocrystalline anisotropy has been assumed for the discrepancy.

We also studied the size dependence of coercivity. At finite temperatures, the thermal fluctuation reduces the threshold field (a type of super-paramagnetism). We have confirmed that such reduction saturates when the size reaches 20 nm, and thus the above estimate is essentially valid up to a grain size of a few hundred nanometers. However, for larger grain sizes, the reduction due to DDI increases. Such dependence is also studied in Section 5. When the grain size further increases, DDI induces the formation of a multidomain magnetic structure in a grain [43]. In such a system, the mechanism of nucleation may be different from that of a nanosize grain. We systematically studied the dependence of magnetic patterns in large flat systems on parameters (anisotropy energy, DDI) and obtained a phase diagram of magnetic patterns [44]. To study the metastability of the uniformly magnetized state in such a large system whose stable state is a multidomain magnetic structure, we compared the phase diagrams obtained by thermal-quench process corresponding to the thermal demagnetization process and by field-quench process corresponding to the remanence process, and found metastability in some regions in the phase diagram. We also found that the nucleation breaking the uniformly magnetized state occurs in the middle of the surface owing to DDI effects, in contrast to the case without DDI, in which it occurs from the corners.

For the coercivity of realistic polycrystalline magnets, namely, an ensemble of grains, the interaction among the grains plays an important role. We have studied the effect of a grain boundary phase consisting of a soft magnet and also the threshold field of domain wall pinning in a sandwich (hard-soft-hard magnet) configuration [45–51]. We also report effects of modifications of surface anisotropy on the coercivity [52]. The effects of misalignment are also important when studying coercivity. Fujisaki et al. [53] studied the alignment dependence of coercivity using the LLG equation and explored the possibility of the Kondorsky dependence 1/cosθ on the field direction. This problem was studied in detail by Bance et al. [54]. The effects of the attachment of a soft-magnet boundary phase have been studied by micromagnetic simulation with temperature-dependent parameters [24,27].

Moreover, the ensemble effects of grains are also important. An ensemble of independent grains (a hysteron) with a distribution of their coercive fields is modeled by the so-called Preisach model [55]. Each grain has a hysteresis curve depending on the value of the field at which the field is swept back (first-order reversal curve (FORC)) [56]. The distribution of the coercive fields is related to the FORC by a mathematical formula, and the distribution is called the FORC diagram. The interaction among grains causes changes in the diagram, and the FORC diagram is used to classify the nature of magnets [57], and it is even used to classify earthenwares in archaeology, because they contain magnetic particles [58]. To study this effect, extensions of Preisach model have been explored [59,60].

The rest of the paper consists of the following: In Section 2, the model used in the present paper is explained. In Section 3, the thermodynamic properties and methods used are presented. In Section 4, approaches to examining the coercivity of nanoparticles by the SLLG and free energy methods are given. In Section 5, the coercivity of large grains where DDI is relevant is studied. In Section 6, the effects of the grain boundary and surface properties are studied. In Section 7, the coercivity of an ensemble of grains is discussed. In Section 8, the summary and perspective are given. In Appendix A, we briefly explain the exchange couplings and magnetic moments that we estimated for the parameters of the Hamiltonian.

## 2. Model

We adopt the following atomistic Hamiltonian for the Nd magnet [23]:

(2) $$H=-\sum_{i<j}\;2J_{ij}s_{i}\cdot \;\;s_{j}-\sum_{i}^{Fe}D_{i}(s_{i}^{z}{)}^{2}+\sum_{i}^{Nd}B_{l,i}^{m}{\hat{O}}_{l,i}^{m}-h\sum_{i}S_{i}^{z},$$

where $s_{i}$ denotes the classical spin at the ith site and $|\;s_{i}|$ depends on the type of site. In a unit cell of Nd${\;}_{2}$Fe${\;}_{14}$B (Figure 1(a)), there are two types of Nd site, six types of Fe site, and one type of B site. $J_{ij}$ is the exchange interaction between the ith and jth sites, $D_{i}$ is the magnetic anisotropy constant for Fe atoms, the third term is the crystal electric field (CEF) for the magnetic anisotropy energy of Nd atoms, and h is the external magnetic field. The CEF for a rare-earth atom (ion) is given in the following form:

(3) $$H_{CEF}=\sum_{l,m}B_{l}^{m}{\hat{O}}_{l}^{m},B_{l}^{m}=\Theta_{l}A_{l}^{m}\langle r^{l}\rangle .$$

Here, ${\hat{O}}_{l}^{m}$ is the Stevens operator in the classical manner, for example, ${\hat{O}}_{2}^{0}=3J_{z}^{2}-J^{2}$. $\Theta_{l}$ and $A_{l}^{m}$ are the Stevens factor and the coefficient of the spherical harmonics of the CEF, respectively. r is the radiation diameter of an electron and $\left\langle r^{l}\right\rangle$ is the average over the radial wave function estimated in Ref [61]. In our works, we adopt the terms of l=2,4,6 with only the diagonal operators (m=0), which give the dominant contribution to the tilt of the spins in the ground state.

**Figure 2.**
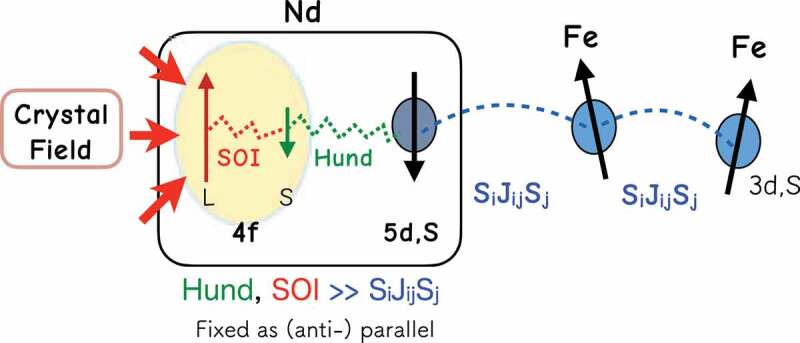
Magnetic interactions in a Nd atom

For Fe and B atoms, $s_{i}$ denotes the magnetic moment at the ith site, but for Nd atoms, $s_{i}$ is the moment of valence (5d and 6s) electrons. For the Zeeman energy, the magnetic moment of site i is given by $S_{i}$. For the Fe and B sites, $S_{i}\;\;=\;\;\;s_{i}$. For Nd, however, not only $s_{i}$ but also the magnetic moment $J_{i}$ of the 4 f electron contributes. Here, $J_{i}$ consists of the orbital moment L and the spin moment $s{\;}_{4f}$, which are antiferromagnetically coupled by the spin–orbit interaction (SOI), and is given by $|\;J_{i}|=g_{T}J\mu_{B}$, in which $g_{T}=8/11$ is the Landé g-factor and J=9/2. $s_{4f}$ and $\;s_{i}$ are ferromagnetically coupled by the Hund rule as schematically depicted in Figure 2 [62]. The total moment for each Nd atom is $S_{i}=\;\;\;s_{i}\;\;+\;\;\;J_{i}$, which is used for the interaction with the magnetic field, while the exchange interaction is given in the form $J_{ij}\;\;s_{i}\cdot \;\;\;s_{j}$ as in (2), which is antiferromagnetic between Fe and Nd. We adopted the values of Stevens coefficients $A_{l}^{m}$ estimated from experimental data [63].

For the anisotropy of Fe, we considered only the single-ion anisotropy $D_{i}$ and adopted the values calculated by Miura et al. [64]. B makes little contribution to the magnetic Hamiltonian.

Nd${\;}_{2}$Fe${\;}_{14}$B is an itinerant magnet. Here, we express the magnetic interaction in the form of the Heisenberg model. Thus, the interaction is widely distributed owing to the itinerancy of electrons. We adopted the exchange energies between spins obtained by first-principles calculation with the Korringa-Kohn-Rostoker (KKR) Green’s function method [65] (Akai-KKR). The interactions between spins are widely distributed as shown in Figure 1(b). In the calculation, we cut the interactions separated by longer than $r_{cut}$. In most of the calculations, we used the interaction up to 3.52 Å. All data for the magnetic moments *s_i_* and exchange couplings $J_{ij}s_{i}s_{j}$ estimated by the Akai–KKR method are given in Supplementary Material 1 with a brief explanation in Appendix A.

## 3. Thermodynamic properties

### 3.1. Macroscopic properties

#### 3.1.1. Magnetizations

**Figure 3.**
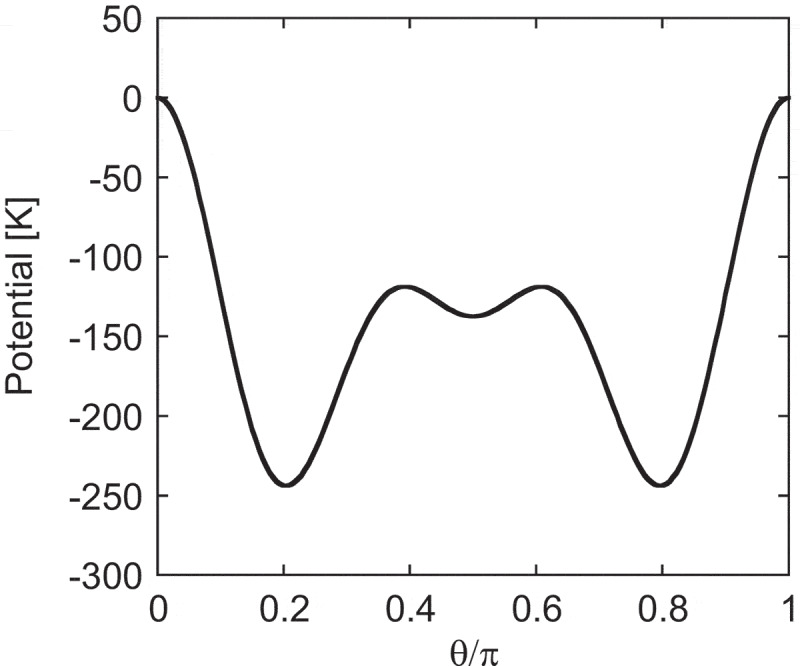
Anisotropy energy $E_{A}(\theta )$ for a Nd atom

First, we study the temperature dependence of magnetization. It is known that the Nd magnet exhibits a spin-reorientation (SR) transition at $T_{r}∼$135 K, below which the magnetization is tilted from the c-axis [7–9,63]. For the tilt in the ground state, the anisotropy energy must have the minimum point at a non-zero angle from the c-axis. Thus, we check the CEF for Nd. Substituting $J_{z}=J\cos\theta$ into (3), the anisotropy energy at 0 K for Nd atoms is expressed with diagonal terms in the following form:

(4) $$E_{A}=K_{1}\sin^{2}\theta +K_{2}\sin^{4}\theta +K_{4}\sin^{6}\theta ,$$

where $K_{1}=-3f_{2}B_{2}^{0}-40f_{4}B_{4}^{0}-168f_{6}B_{6}^{0}$, and so on, with positive constants $f_{l}$. With the coefficients $A_{0}^{2}=295.0$ K a${\;}_{0}^{-2}$, $A_{0}^{4}=-12.3$ K a${\;}_{0}^{-4}$, and $A_{0}^{6}=-1.84$ K a${\;}_{0}^{-6}$ (a${\;}_{0}$ is the Bohr radius) estimated by Yamada et al. [63], the first single-ion anisotropy satisfies $K_{1}<0$ at T=0. Note that although $A_{0}^{2}>0$, and thus $B_{2}^{0}<0$ (∵ the Stevens factor $\Theta_{2}<0$), the contributions of the other terms ($B_{4}^{0}$ and $B_{6}^{0}$ terms) make $K_{1}<0$. This potential energy causes a tilted magnetization in the ground state (at zero temperature) as shown in Figure 3, in which θ≃0.2π gives the minimum.

**Figure 4.**
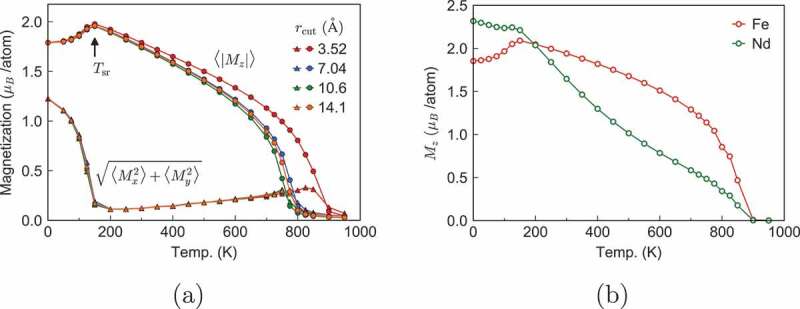
(a)Temperature dependence of magnetizations. Circles and triangles denote $M_{z}$ and $M_{xy}$, respectively. (b) Temperature dependence of magnetizations of Fe and Nd atoms. (From reference [23]: modified.)

In Figure 4(a), the temperature dependences of the z and xy components of magnetization are depicted [23]. The SR transition is clearly observed at the transition temperature $T_{r}≃145$K, which is close to experimentally estimated values (≃135K). $T_{r}=145$K does not depend on the choice of the range of interaction, $r_{cut}$, as explained at the end of Section 2. This fact indicates that at low temperatures around $T_{r}$, the competition between the anisotropy and short-range strong interactions is relevant.

As to the ferromagnetic phase transition, the critical temperature is given as $T_{c}∼$ 750–870 K, depending on $r_{cut}$. These values are slight overestimates compared with the experimental values [4,7] of $T_{c}∼$ 585 K. This difference is attributed to the exchange constants used in the calculation, and we may need to rescale the exchange constants. Nevertheless, the overall properties are semi-quantitatively reproduced and there are no serious problems in studying the effects of the thermal fluctuation. Therefore, we accept the present model. When we compare the results with experimental ones, the temperature rescaled by the critical temperature should be used.

Owing to the atomistic model, we can observe atom-specific properties individually, for example, the temperature dependence of the magnetizations of Fe and Nd atoms as depicted in Figure 4(b). As shown in the figure, the magnetization of Nd decreases much faster than that of Fe as the temperature increases. We attribute this difference to the interactions. Namely, the exchange coupling between Nd and Fe is small, while Fe spins are strongly coupled with each other (the exchange constants between Nd atoms are negligible).

#### 3.1.2. Angle dependence of free energy

**Figure 5.**
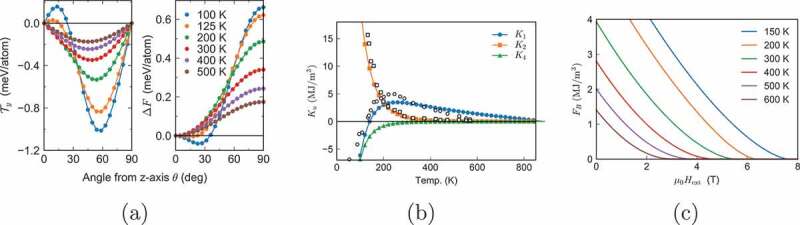
(a)Temperature dependence of torque and excess free energy as functions of angle from the c-axis. (b) Temperature dependence of anisotropy constants $K_{1},K_{2}$, and $K_{4}$. Open circles and squares show the corresponding values obtained experimentally for $K_{1}$ and $K_{2}$ [68]. (c) Temperature dependence of excess free energy as a function $H_{ext}$ at various temperatures. (From reference [23]: modified.)

To obtain the temperature dependence of anisotropy energies $K_{i}(T)$ (4), we need the angle dependence of the free energy F(θ), for which we adopted the constrained Monte Carlo (C-MC) method. In this method, the total magnetization $M\;\;=(\;\;\;M_{x},\;\;\;M_{y},\;\;\;M_{z})$ is fixed in a direction. For a fixed angle θ (the angle between the c-axis and the magnetization), we calculated the magnetization torque T defined by

(5) $$T\;\;=-{\left\langle \sum_{i}^{N}\;\;\;e{\;}_{i}\times \frac{\partial H}{\partial e_{i}}\;\right\rangle }_{MC},$$

and using it, the excess free energy ΔF(θ)=F(θ)−F(0) is given by

(6) $$\Delta F(\theta )=\int_{0}^{\theta }d\theta {\;}^{\prime }\left[n\;\;(\theta {\;}^{\prime })\;\;\times \;\;T\;\right]\cdot {\left.\frac{\partial n\;(\theta {\;}^{\prime })}{\partial \left(\theta {\;}^{\prime }\right)}\right|}_{\theta =\theta {\;}^{\prime }},$$

where $e_{i}$ and n are the unit vectors of $s_{i}$ and M, respectively [66]. In Figure 5(a), the obtained angle dependence of the torque and the free energy are given. By analyzing the angle dependence of the free energy using the form in (4), the temperature dependences of the coefficients $K_{1}(T),K_{2}(T)$, and $K_{4}(T)$ are obtained (Figure 5(b)), which agree with those in previous works [67,68].

Using the angle dependence of the free energy on the magnetic field $H_{ext}$, we obtain free energies as a function $H_{ext}$ at various temperatures as depicted in Figure 5(c), which gives an idea for the temperature dependence of the energy barrier for the uniform (coherent) rotation of magnetization [23,69]. However, a more comprehensive study on the temperature dependence of coercivity will be given in Section 4.2.

### 3.2. Domain wall

**Figure 6.**
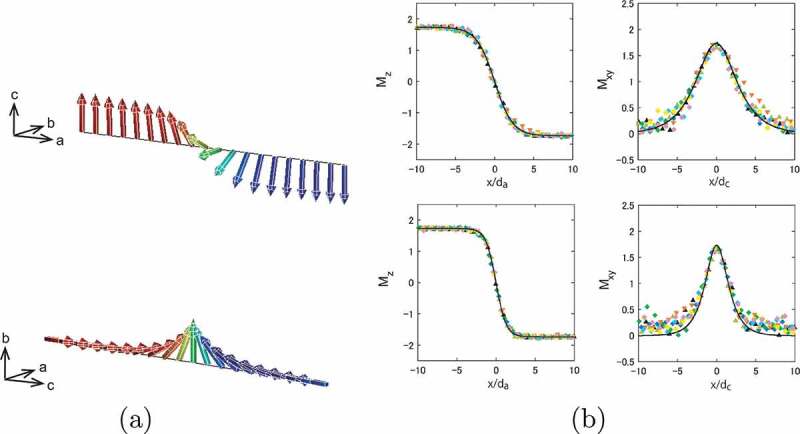
(a) (top) Domain wall propagating along the a-axis (type I, Bloch-type wall), (bottom) domain wall propagating along the c-axis (type II, Néel-type wall). (b) (top left) $M_{z}$ along the a-axis (type I) at 300 K. The unit of the vertical axis is $\mu_{B}$/atom. (top right) $M_{xy}$ along the a-axis (type I) at 300 K. (bottom left) $M_{z}$ along the c-axis (type II) at 300 K. (bottom right) $M_{xy}$ along the c-axis (type II) at 300 K. The analytical functions $m_{z}(x)$ and $m_{y}(x)$ are given by black lines. Here symbols denote $M_{z}$ ($M_{xy}$) at different Monte Carlo steps. (From reference [25]: modified.)

The profile of a magnetic domain wall is one of the important characteristics of magnets. We studied the profile of the domain wall of the Nd magnet and showed how well the present atomistic model reproduces the microscopic ordering property [25,70]. Because of the anisotropy of the crystal structure, the profile depends on the direction. Along the a-axis (perpendicular to the easy axis), the domain wall is of the Bloch type, while it is of the Néel type along the c-axis, as depicted in Figure 6(a). In the case of the Bloch type propagating along the x-axis, the z component of magnetization shows a step-like structure and the y component shows a bell-type structure,

(7) $$m_{z}(x)=-m(T)\tanh\left(\frac{x}{\delta_{0}}\right)$$

and

(8) $$m_{y}(x)=m(T)\cosh^{-1}\left(\frac{x}{\delta_{0}}\right),$$

respectively, and $m_{x}(x)=0$. Here, $\delta_{0}$ is the wall parameter

(9) $$\delta_{0}\equiv \sqrt{\frac{A}{K_{1}}},$$

and the width of the domain wall is given by $\xi =\pi \delta_{0}$. These dependences also apply to the Néel type. The profiles in both cases at T=300K are depicted in Figure 6(b). The profiles are well fitted by (7) and (8). The width of the domain wall is consistent with the experimentally estimated value [71]. We found that the width of the Bloch type (along the a-axis) is larger than that of the Néel type (along the c-axis). This difference originates from the differences in the stiffness constants with the direction, which we study in more detail in Section 3.3. We obtained the width for various temperatures and found that it increases with the temperature, indicating that the renormalization of the stiffness constant is faster than that of the anisotropy energy.

### 3.3. Anisotropy of exchange stiffness constant

**Figure 7.**
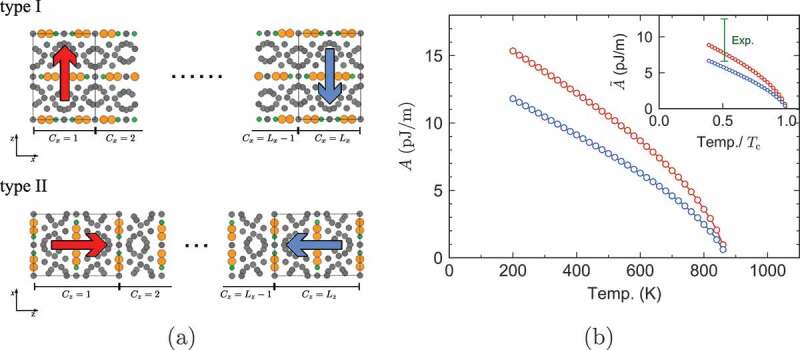
(a) Domain wall propagating along the a-axis (type I) and domain wall propagating along the c-axis (type II). (b) Temperature dependence of exchange stiffness constants, A, for the DW type I (red circle) and type II (blue circle). Inset shows the renormalized values, $\tilde{A}$, and the green bar denotes the range of the experimental values at room temperature. (From reference [24]: modified.)

Because of the anisotropy of the crystal, the exchange stiffness depends on the direction as depicted in Figure 7(a), which causes the difference in the domain wall shape as mentioned in Section 3.2 [25]. We explicitly studied the dependence of the stiffness constants $A_{x}(T)$ along the a-axis and $A_{z}(T)$ along the c-axis [24].

To obtain the stiffness constants at a given temperature, we used domain wall energy $E_{dw}(T)$ and the anisotropy energy $E_{K}(T,\theta )$ [72,73]. The former is expressed in the continuum model as

(10) $$E_{dw}(T)=2\sqrt{A(T)}\int_{0}^{\pi }d\theta \;\sqrt{E_{K}(T,\theta )}.$$

We regard $E_{dw}(T)$ as equal to the domain-wall free energy in the atomistic spin model, $F_{dw}(T)$, which is given by

(11) $$F_{dw}(T)=T\int_{T}^{\infty }dT^{\prime }\;\frac{E_{dw}(T^{\prime })}{{\left(T^{\prime }\right)}^{2}},$$

where $E_{dw}(T)$ is the internal energy of domain-wall formation, which is defined as the difference between internal energies for systems with a parallel periodic condition and an antiparallel periodic boundary condition. The anisotropy energy $E_{K}(T,\theta )$ was calculated by the C-MC as in Section 3.1.2. The thus obtained temperature dependence of the stiffness constants along the a-axis ($A_{x}(T)$) and c-axis ($A_{z}(T)$) is depicted in Figure 7(b). Gong et al. have obtained a similar direction dependence of the stiffness constants and extended the study to the interface exchange coupling strength between the Nd magnet and the grain boundary phase [28].

Recently, the anisotropy of the stiffness constants has been evaluated by spin-wave measurement in a single crystal sample [74], where the anisotropy is weaker than the above result at high temperatures. This difference may be due to how we define the exchange stiffness constant and/or the existence of long-range exchange interactions and so on. This difference must be clarified in the future.

The method used in our approaches is general and can be applied for other systems. For example, similar calculations have also been carried out for the magnets SmCo${\;}_{5}$ [75] and SmFe${\;}_{12}$ [76]. $A_{x}(T)∼A_{z}(T)$ for the former and $A_{x}(T)<A_{z}(T)$ for the latter, which suggest significantly different anisotropic properties from Nd${\;}_{2}$Fe${\;}_{14}$B.

### 3.4. FMR

The dynamics of magnetization is simulated by the LLG equation. To take into account the thermal fluctuation, we used the SLLG equation 21,22 in which we add a white Gaussian random field $\left\{\xi_{i}\right\}$ to the LLG equation:

(12) $$\frac{d}{dt}S_{i}=-\frac{\gamma }{1+\alpha_{i}^{2}}S_{i}\times (H_{i}^{eff}+\xi_{i})-\frac{\alpha_{i}\gamma }{(1+\alpha_{i}^{2})S_{i}}S_{i}\times (S_{i}\times (H_{i}^{eff}+\xi_{i})),$$

where $\alpha_{i}$ is the damping factor at the ith site, γ is the gyromagnetic constant, and

(13) $$H_{i}^{eff}=-\frac{\partial H}{\partial S_{i}}$$

is the effective field due to the exchange interaction and the anisotropy terms. We adopted the commonly accepted value of $\alpha_{i}=0.1$ for the damping constant [17]. The random field $\xi_{i}$ satisfies

(14) $$\langle \xi_{k}^{\mu }(t)\rangle =0,\;\langle \xi_{k}^{\mu }(t)\xi_{l}^{\nu }(s)\rangle =2{\tilde{D}}_{k}\delta_{kl}\delta_{\mu \nu }\delta (t-s),\;\mu =x,y,\;or\;z.$$

The strength of the noise, ${\tilde{D}}_{k}$, is related to the temperature of the system by the fluctuation–dissipation relation

(15) $${\tilde{D}}_{i}=\frac{\alpha_{i}}{S_{i}}\frac{k_{B}T}{\gamma }.$$

The physical noise has a finite auto-correlation time, and the white-Gaussian noise whose auto-correlation time is zero is an extreme case for short correlation noise. Indeed, the white-Gaussian noise contains very high-frequency components which should be quantum mechanically suppressed at a finite temperature. Such situation has been studied in the literature [77]. However, it is also known that in the classical limit ℏ→0, the present treatment is consistent, and the present SLLG realizes the dynamics of probability distribution of the Fokker–Planck equation which leads the distribution to the thermal equilibrium distribution at a given temperature [21,22]. Thus, as a classical model for such process, we adopt the present scheme of dynamical model.

Using the Kubo formula [29,78], we obtained the temperature dependence of the FMR resonance frequency at zero external field from the time-correlation function $C^{\alpha }(\tau )$ (α=x,y,orz) of magnetization $\;M\;\;=(M^{x},M^{y},M^{z})$ in equilibrium. The FMR spectrum I(f) is given by the time-correlation function $C^{\alpha }(\tau )$ as

(16) $$I^{\alpha }(f)\equiv \frac{1}{T}\int_{t_{0}}^{t_{0}+T}d\tau C^{\alpha }(\tau )e^{i2\pi f\tau },$$

where

(17) $$C^{\alpha }(\tau )=\frac{1}{T}\int_{t_{0}}^{t_{0}+T}dt\langle M^{\alpha }\left(t\right)M^{\alpha }\left(t+\tau \right)\rangle .$$

The time-correlation function was obtained by the SLLG method.

**Figure 8.**
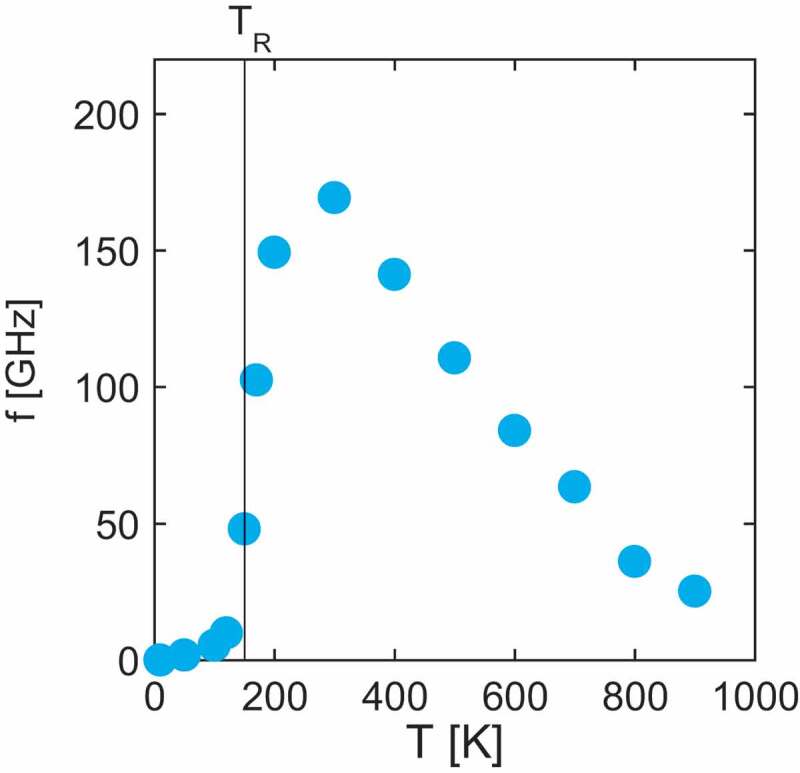
Temperature dependence of resonance frequency $f_{R}$ for the Nd magnet model. (From reference [29]: modified.)

In Figure 8, we depict the temperature dependence of the resonance frequency of the Nd-magnet model. Here, we find that the peak of the spectrum shows a nonmonotonic dependence on the temperature and that the almost zero FMR frequency in the low-temperature phase reflects SR transition [29].

Now we study why the resonance frequency is zero, $f_{R}∼0$, in the low-temperature phase. This property is related to the tilted configuration. Here, we consider the dynamics of the total magnetization M of a minimal model for SR, that has exchange interactions and the first and second anisotropy energies (18), because the spins are tightly connected by the interaction and they move together. FMR is the response of the total magnetization to the external uniform field. The effective field for the precession motion applied to the total magnetization is given as

(18) $$\begin{array}{rl} & H^{eff}=-\frac{\partial H_{Aniso}}{\partial M},\\ & H_{Aniso}=-D_{1}\sum_{i}{\left(M_{z}\right)}^{2}-D_{2}\sum_{i}{\left(M_{z}\right)}^{4}, \end{array}$$

where $H_{Aniso}$ is the contribution from the anisotropy term of the minimal model. The precession frequency is given by

(19) $$f=\gamma h_{eff}/(2\pi ),\;h_{eff}=|\;H^{eff}|.$$

For $D_{2}=0$, the resonance frequency is given by $f_{R}=\gamma h_{eff}/(2\pi )=2D_{1}M_{z}/(2\pi )$. Thus, $f_{R}$ is proportional to $M_{z}$, which is the conventional temperature dependence in the usual FMR.

On the other hand, for $D_{2}\neq 0$, the situation is different. Considering the relations

(20) $$\frac{dE_{G}}{d\theta }{|}_{\theta =\theta_{0}}=\frac{dE_{G}}{dM_{z}}\frac{dM_{z}}{d\theta }{|}_{\theta =\theta_{0}}=-h_{eff}\sin\theta_{0},$$

we note the relation

(21) $$h_{eff}=0,$$

because the following relation holds at the tilted configuration:

(22) $${\left.\frac{dE_{G}}{d\theta }\right|}_{\theta =\theta_{0}}=0,\;\sin\theta_{0}\neq 0.$$

Thus, we have an important consequence:

(23) $$f=\gamma \frac{h_{eff}}{2\pi }=0\;for\;\theta =\theta_{0}\neq 0.$$

In the present calculation, anisotropy in the plane given by Stevens operators with m≠0, is not taken into account. If non-zero m terms exist, the effective field deviates from the z-axis and the resonance frequency is not zero. However, non-zero terms are small, and the large reduction in resonance frequency around the SR transition temperature obtained here (Figure 8) is one of the characteristics of the present material.

## 4. Coercivity of nanoparticles

**Figure 9.**
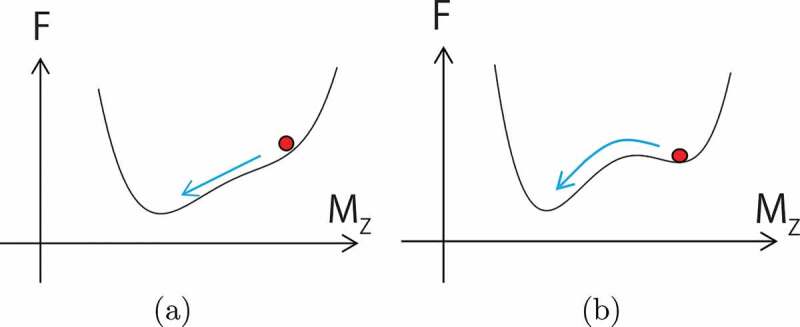
(a) Deterministic process. (b) Stochastic process by barrier-crossing dynamics

Coercivity is a threshold field for metastable magnetization, and the situation is schematically depicted in Figure 9. Coercivity at zero temperature is given by the field at which the barrier disappears. However, at finite temperatures, the thermal fluctuation causes a jump over the barrier as shown in Figure 9(b). In the absence of a barrier (Figure 9(a)), magnetization relaxes smoothly in a deterministic manner. On the other hand, in the case of Figure 9(b), relaxation is triggered by a large thermal fluctuation and occurs stochastically as a type of Poisson process, where the distribution of the relaxation time is large.

Magnets consist of grains, and magnetization reversal is a sequence of reversals of grains. Thus, as a fundamental process, we first studied the process in a single grain. At zero temperature, the reversal occurs as the Stoner-Wohlfarth process [79]. However, at finite temperatures, the effect of thermal agitation plays an important role [20]. To study this effect quantitatively, we adopted two complementary methods, that is, a direct SLLG simulation of the dynamics of magnetization [38] and an analysis of free energy as a function of magnetization F(M) obtained by a MC simulation [40].

### 4.1. Dynamics of magnetization

**Figure 10.**
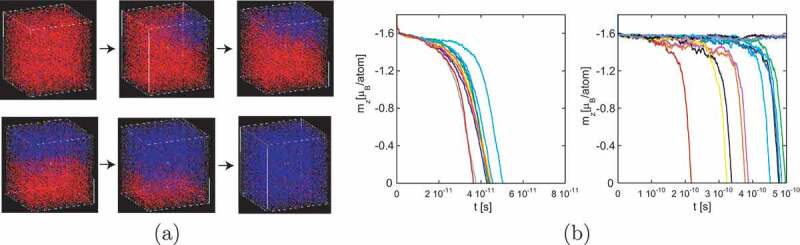
(a) Snapshots of the magnetization reversal from the all-down spin state under a reversed field (h = 4.0 [T]). Red and blue arrows denote down-spin and up-spin states, respectively. (b) Examples of time evolutions of magnetization relaxation curves at (left) h = 8 T and (right) h = 4.1 T. α= 0.1. (From reference [38]: modified.)

By using the SLLG equation (12), we studied the dynamics of magnetization. In Figure 10(a), we show snapshots of the magnetization reversal from the down-spin state for α=0.1 under a reversed field, h=4.0 T, which is in the stochastic region. There, we find that nucleation occurs from a corner. Then, the reversed region expands first in the ab plane by a Bloch-type domain wall and then grows in the direction of the c-axis by a Néel-type domain wall. We observed that this tendency is independent of α. This process is attributed to the fact that the effective exchange interactions along the a- and b-axes are stronger than that along the c-axis [24,25].

In Figure 10(b), we depict the time dependence of the magnetization $m_{z}=\sum_{i=1}^{N_{site}}S_{i}^{z}/N$ in the relaxation processes for α=0.1 at h=8T (Figure 10(b) (left)) and at h=4.1T (Figure 10(b) (right)).

There are two typical types of relaxation, that is, deterministic and stochastic. The former type occurs for a large field, and the relaxation is characterized by a multi-nucleation Avrami process. Examples of relaxation (12 samples) of magnetization are shown in Figure 10(b) (left), where the distribution of relaxation times is small. On the other hand, the latter type occurs for a small field, and the relaxation is characterized by a single nucleation. In this case, the distribution of relaxation times is very wide as shown in Figure 10(b) (right). There, a few samples do not relax, and thus it is impossible to estimate the average relaxation time until they relax. At the border between the two cases, the relaxation time increases very rapidly, and this region of the field can be regarded as the practical end of metastability, which is called the dynamical spinodal point [80].

In the latter case, the large distribution of relaxation times prevents us from estimating the average relaxation time. To overcome this difficulty, we introduced a statistical method to evaluate the relaxation time. We derived the statistical relation between the reversal probability p and the relaxation time τ. If an event (relaxation) occurs with the probability p in a unit time, the probability that the event occurs for the first time in the period [t;t+Δt] is $pe^{-pt}\Delta t.$ The mean relaxation time ⟨τ⟩ is given by

(24) $$\langle \tau \rangle =p\int_{0}^{\infty }te^{-pt}dt=\frac{1}{p}.$$

**Figure 11.**
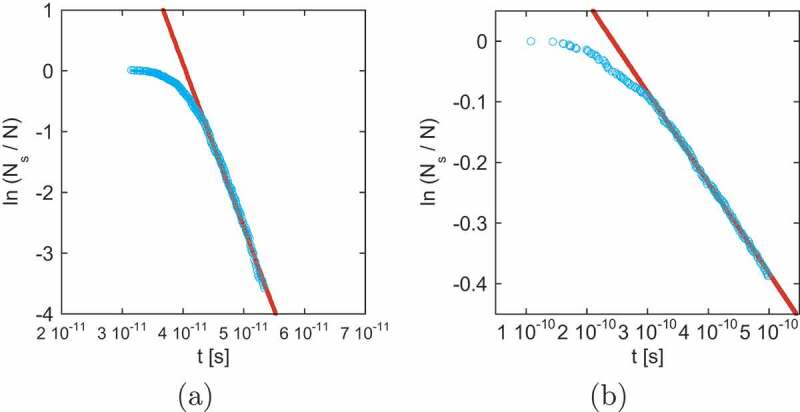
Time dependence of $\ln(N_{s}/N)$. Blue circles denote time dependence of $\ln(N_{s}/N)$ at (a) h = 8 T and (b) h = 4.1 T. α = 0.1. For (a) and (b), the slopes $p=2.697\times 10^{11}$s${\;}^{-1}$ and $p=1.491\times 10^{9}$s${\;}^{-1}$ are estimated respectively by linear fitting (red lines). Details are given in text. (From reference [38]: modified.)

The probability P(t) that the event occurs in the period [0,t] is $P(t)=1-e^{-pt}.$ If we perform N simulations, the number of surving (unchanged) samples is $N_{sv}(t)=N-N_{done}(t)=Ne^{-pt}.$ Then, p (and τ) can be estimated from the slope of $\ln(N_{sv}(t)/N)$ versus −pt. We plot $\ln(N_{sv}(t)/N)$ as a function of t in Figure 11. From the slope, we can estimate ⟨τ⟩. In this method, we can recognize the time range of linear dependence where the dynamics is governed by a single nucleation process, which is difficult when taking the naive average of the samples.

**Figure 12.**
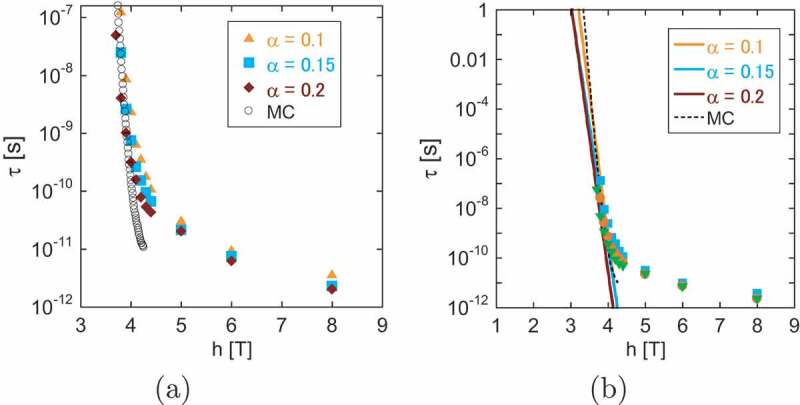
(a) Magnetic field dependence of the relaxation time (magnetization reversal time) on damping factor α. Open circles denote the relaxation time of the Arrhenius law ($\tau =\tau_{0}e^{\Delta F}$), in which ΔF is taken from the Monte Carlo study for $L_{x}=10.6$nm in Figure 14. (b) Extrapolation of the relaxation time to estimate the field at which the relaxation time is 1s (coercivity) for different values of damping factor α. (From reference [38]: modified.)

In Figure 12(a), we give the field dependence of the relaxation time with different α values. The relaxation time increases rapidly below h≃4.2T. For large relaxation times, we expect the single exponential decay of the Arrhenius type. Thus, when we extrapolate the increasing relaxation times, we fit them including a correction term in the form of a double exponential fitting,

(25) $$\tau (h)=Ae^{-ah}+Be^{-bh}=Ae^{-ah}\left(1+Ce^{-dh}\right),$$

where C=B/A and d=b−a. In Figure 12(b), the fitted curves of Eq. (25) are plotted for different α values. The intersection of each line with τ=1s gives coercivity. The estimated coercivities for α=0.1, 0.15, and 0.2 are $h_{c}≃3.2$, 3.0, and 3.0, respectively, and we find that coercivity is

(26) $$h_{c}≃3T.$$

This value is close to the estimation $h_{c}≃3.3$ obtained by the MC method [40], which will be reviewed in the next subsection. The dashed line in Figure 12(b) is the estimation by the MC method. We find that, although the simulation time of the SLLG method is limited and much shorter than 1s, owing to the fact that the relaxation is governed by a single nucleation process, the extrapolation of the relaxation time using τ(h) as a fitting function is effective for estimating coercivity.

### 4.2. Monte Carlo method

#### 4.2.1. Free energy as a function of magnetization

The free energy for a given temperature T and a field H is given by

(27) $$F(T,H)=-k_{B}T\ln Z(T,H),Z(T,H)=Tre^{-\beta H_{0}-\beta H\sum_{i}^{N}S_{i}^{z}},$$

where $H_{0}$ is the Hamiltonian without the field, $S_{i}^{z}$ is the spin at the ith site, and $\sum_{i}^{N}S_{i}^{z}$ is the total magnetization. If we fix the magnetization to M, then

(28) $$F(T,H;M)=-k_{B}T\ln Z(T,H;M),Z(T,H;M)=Tre^{-\beta H_{0}-\beta H\sum_{i}^{N}S_{i}^{z}}\times \delta (\sum_{i}^{N}S_{i}^{z}-M).$$

The probability that the system has the magnetization M is given by

(29) $$P(M)=\frac{Z(T,H;M)}{Z(T,H)},$$

and hus F(T,H;M) can be obtained from the distribution function of M as

(30) $$F(T,H;M)-F(T,H;0)=-k_{B}T\ln P(M)P(0).$$

In principle, the distribution can be obtained from a histogram of M via MC simulation. However, for large systems the ratio P(M)/P(0) is $e^{aN}$, where a is a constant of order O(1). MC simulation does not produce states for which P(M) is very small. Thus, it is practically impossible to obtain P(M) for the entire range of M. Wang and Landau proposed a method of overcoming this difficulty [41]. We used the method and obtained P(M) in the case H=0, and obtained F(T,0;M), from which F(T,H;M) is obtained easily by the relation

(31) F(T,H;M)=F(T,0;M)−HM.

**Figure 13.**
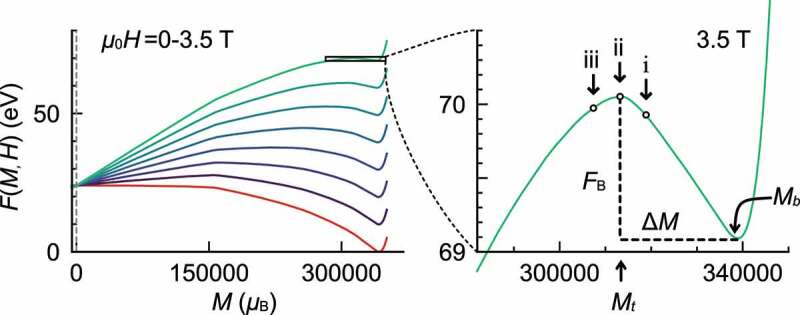
(left) Free energies as a function of M of the Nd${\;}_{2}$Fe${\;}_{14}$B isolated grain whose size is $(L_{x},L_{y},L_{z})=$ (14.1,14.1,14.6)nm (212,536 spins). $T=0.46T_{C}^{cal}$. Red line is F(T,H=0;M), and other lines are for those with H>0. (right) magnified F(T,H;M) around the metastable state. (From reference [40]: modified, © 2020 The Authors)

**Figure 14.**
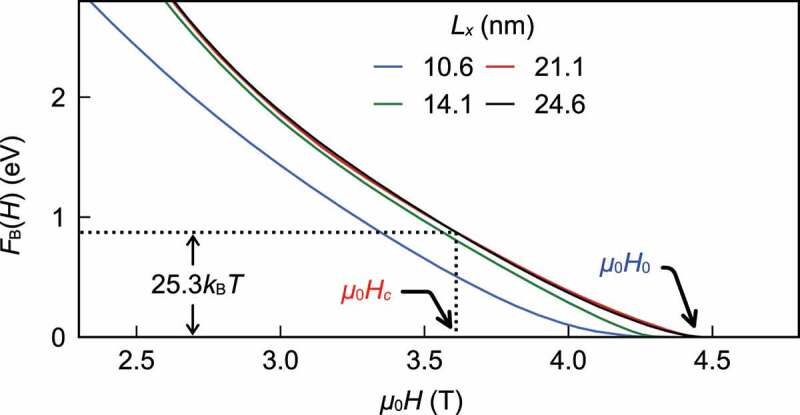
Free energy barriers as a function of $\mu_{0}H_{z}$ for four system sizes: $L_{x}=10.6\;nm$, 14.1nm, 21.1nm, and 24.6nm ($L_{y}=L_{x}$, $L_{z}=1.038L_{x}$.) (From reference [40]: modified, © 2020 The Authors)

In Figure 13(left), F(T,H;M) is depicted for several fields. The free energy barrier $F_{B}$ is defined as depicted in Figure 13(right). The field dependences of the free energy barrier $F_{B}(H)$ for several sizes are given in Figure 14. Here we find that the size dependence of F(T,H;M) saturates for L>20nm, and they are large enough to study the cases of larger sizes. Here, we define $H_{0}$ as the field where $F_{B}$ becomes zero, at which the magnetization becomes unstable. The relaxation time is zero at this field. At finite temperatures, the metastable state may relax with thermal fluctuation. Using the Arrhenius model, we estimate the rate of relaxation per 1s and the relaxation time τ as

(32) $$r=N_{contact}e^{-\beta F_{B}},\;\tau =\frac{1}{r}=\frac{e^{\beta F_{B}}}{N_{contact}},$$

where $N_{contact}$ is the number of contacts with the thermal bath in 1s, which is usually given as $10^{11}$. Thus, for the relaxation time of 1s,

(33) $$F_{B}=k_{B}T\times \ln(10^{11})=25.3k_{B}T.$$

The field that gives this value is the coercivity for the relaxation time of 1s, which we call the thermally activated coercivity $H_{c}$.

**Figure 15.**
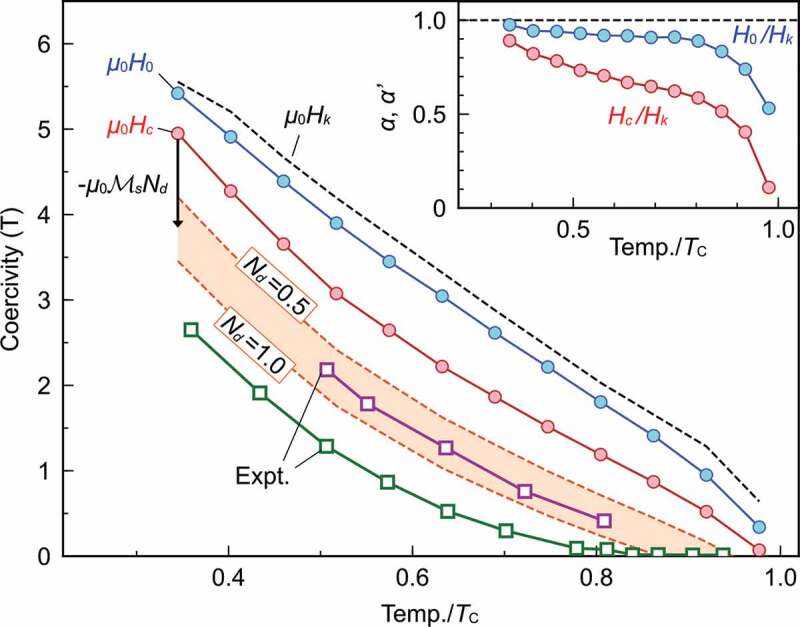
Temperature dependence of coercivity. Blue line $\mu_{0}H_{0}$ and red line $\mu_{0}H_{c}$ were calculated from Figure 14 for 21.1nm×21.1nm×21.9nm (713,172 spins) isolated grain at each temperature. The colored area depicts the coercivity $\mu_{0}H_{c}$ under the demagnetization fields in the range of demagnetization factor $N_{d}=$0.5–1.0. Green and purple squared lines denote the experimental measurements in a sintered magnet [81] and a hot-deformed magnet with grain boundary diffusion of Nd-Cu alloy [42], respectively. Inset shows $\alpha =H_{0}/H_{k}$ and $\alpha^{\prime }=H_{c}/H_{k}$. (From reference [40], © 2020 The Authors)

The thus obtained $H_{0}$ and $H_{c}$ at different temperatures are plotted in Figure 15 by blue and red circles, respectively. In the figure, the coercivity $H_{k}$, which is obtained as $H_{0}$ in the system with the periodic boundary condition, is plotted by a dashed line. In the calculated temperature range, we confirmed that $H_{k}$ takes almost the same value as the magnetic anisotropy field $H_{a}=2K_{1}/M_{s}$, where $K_{1}$ is the magnetic anisotropy constant [23,24] and $M_{s}$ is the saturated magnetization at the given temperature. In the figure, experimentally observed coercivities for a sintered magnet [81] and a hot-deformed magnet with the grain boundary diffusion of Nd-Cu alloy [42] are also plotted. Within a nanosize grain, DDI is not relevant. However, when we compare the results with the experimental data of a magnet, we must consider the effect of DDI from other grains as a demagnetization field. The demagnetization field $-N_{d}M_{s}$ approximately represents the effects of DDI as an external uniform field. Here, we do not explicitly consider the size and shape dependences [82] of the demagnetization field, but they are expected in the range of $N_{d}\mu_{B}=$0.5–1.0. If we take into account the contribution of this demagnetization effect, $H_{c}$ gives a good agreement with that of the hot-deformed magnet in which the grains are rather isolated.

The temperature dependence of the coercivity $H_{coercivity}$ has been expressed in the following forms, known as the Kronmüller equation (5,^33^)

(34) $$H_{coercivity}=\alpha H_{k}-H_{t}-M_{s}N_{d}=\alpha^{\prime }H_{k}-M_{s}N_{d},$$

which indicates how much the coercivity is reduced from $H_{k}-M_{s}N_{d}$, which is naively expected. In the first form, $H_{t}=H_{0}-H_{c}$ is the thermal activation field and the parameter α is given by $H_{0}/H_{k}$, while in the second form, all the thermal effects are included in the parameter $\alpha^{\prime }=H_{c}/H_{k}$. Note that α is not the damping factor in the LLG equation. Our approach can handle the temperature dependences of α and $H_{t}$ explicitly. In the inset of Figure 15, the temperature dependences of these parameters are depicted. For some sintered polycrystalline magnets (corresponding to the green line in Figure 15), Kronmüller and Durst phenomenologically estimated the decay factor as $\alpha_{exp}^{\prime }=$ 0.89–0.93 from magnetic properties measured around room temperature [5], which is close to our estimation.

#### 4.2.2. Activation volume

Next, we consider the mechanism of thermal activation (nucleation) for which the concept of ‘activation volume’ has been introduced [32–34,42]. ΔM represents the difference in magnetization between the local minimum $M=M_{b}$ and the local maximum $M=M_{t}$ of the free energy (see Figure 13(right)), i.e. $\Delta M=M_{b}-M_{t}$.

The activation volume has been defined by

(35) $$V=-\frac{1}{\mu_{0}M_{s}}\frac{\partial F_{B}}{\partial H}.$$

By differentiating $F_{B}$

(36) $$F_{B}(H)=F(M_{t},H)-F(M_{b},H)=F(M_{t},0)-F(M_{b},0)+\mu_{0}H(M_{t}-M_{b}),$$

it is obvious that (35) gives the relation

(37) $$V=\Delta M/M_{s}.$$

Here, we note that $F(M_{t(b)},0)$ depends on H since $M_{t(b)}$ is a function of H, but because dF/dM=0 at $M=M_{t(b)}$, the contribution from these terms is zero.

**Figure 16.**
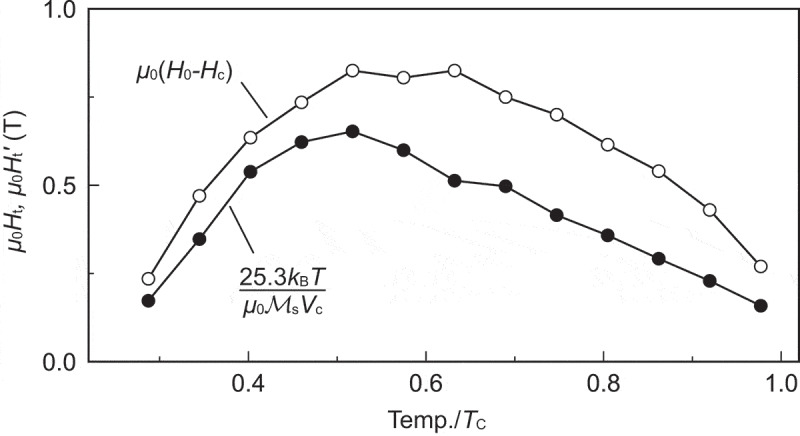
Temperature dependence of the thermal activation reductions of coervity evaluated from the two ways: $H_{t}=H_{0}-H_{c}$ and Eq. (39). (From reference [40], © 2020 The Authors)

If we assume a linear dependence of the barrier on H, that is, taking n=1 in the phenomenological formula

(38) $$F_{B}\propto {\left(1-\frac{H}{H_{0}}\right)}^{n},$$

then we have the widely used phenomenological equation for thermal activation effects [33]:

(39) $$H_{t}^{\prime }=\frac{25.3k_{B}T}{\mu_{0}M_{s}V_{c}},$$

where $V_{c}$ is V at $H=H_{c}$. In Figure 16, we compare the temperature dependences of $H_{t}$ and $H_{t}^{\prime }$. We found a qualitatively similar dependence. The difference between $H_{t}^{\prime }$ and $H_{t}$ becomes significant in the high-temperature range, which is attributed to the fact that the activation volume and n are not exactly constant.

## 5. Coercivity of large grains

**Figure 17.**
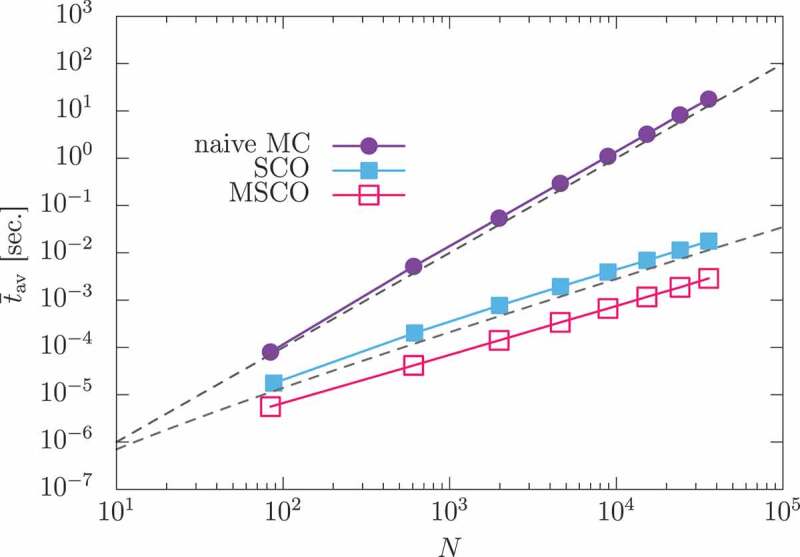
Average computational time $t_{av}$ at T=400K as a function of the number of spins. Violet solid circles, blue solid squares, and magenta open squares indicate the computational time for the naive MC simulation, the SCO method, and the MSCO method, respectively. Upper and lower black dashed lines are proportional to $N^{2}$ and NlnN, respectively. (From reference [44]: modified.)

Because of the uniform magnetization, DDI

(40) $$V_{DDI}(s_{i},s_{j})=D(\frac{s_{i}\cdot s_{j}}{r_{ij}^{3}}-3\frac{\left(s_{i}\cdot r_{ij})(s_{j}\cdot r_{ij}\right)}{r_{ij}^{5}})$$

has a relevant effect on breaking down the uniform ordering. We have studied the effect of DDI for a thin film of the present material. Because DDI is a long-range interaction, the computational cost of simulation increases with the square of the number of spins (N). Various methods have been proposed to overcome this difficulty [83], but they are not very efficient for systems with large unit cells, such as the present material as above. For example, in the method using fast Fourier transform (FFT) a system containing 68 atoms in a unit cell requires 68 modes in Fourier space. The so-called stochastic cutoff (SCO) method has also been proposed as an alternative [84,85]. The SCO method introduces a selection of bonds by the so-called switching procedure. The selection procedure is performed stochastically, maintaining the detailed balance condition. Thus, the stationary state of the simulation is guaranteed to be the same as the equilibrium state of the original model. Because the bond update process rarely adopts long-distance weak bonds, the overall computational time is markedly decreased. As an example, for a three-dimensional system with DDI, one MC step can be computed in a time of O(βNlnN), where N denotes the number of spins in the system. In this method, the complex unit cell also introduces bothersome procedures. In this simulation, we developed a modified SCO method by using of the walker’s algorithm [30]. In Figure 17, we demonstrate that the computational time decreases from $O(N^{2}$) to O(NlnN), and that the modified SCO (MSCO) method gives a smaller coefficient than the conventional SCO method.

### 5.1. Effect of DDI on coercivity of nanoparticles

Thus far, we have studied coercivity without DDI. When the system size increases, DDI becomes an important factor determining coercivity. In this subsection, we study the effect of DDI on the coercivity of nanoparticles using the method of free energy, while the effect of DDI for larger grains in which multiple magnetic domains appear will be studied in Section 5.2.

**Figure 18.**
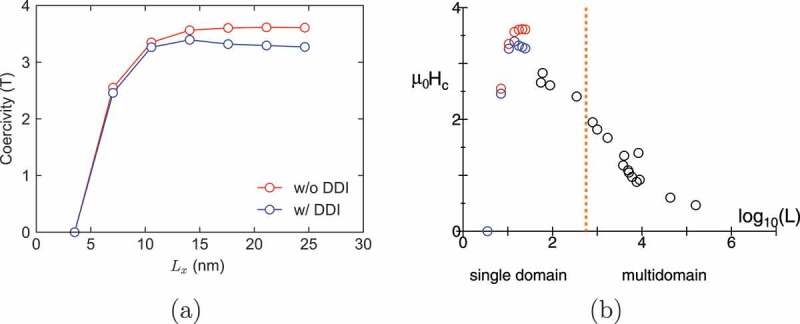
(a) Size dependence of coercivity with and without DDI. Blue and red circles denote the thermally activated coercivity with and without DDI, respectively. (b) Size dependence of coercivity (black circles) taken from literatures [86–89] adding to the data in (a). Above the dotted line, the system tends to have a magnetic multidomain structure

As long as the nucleation starting from corners dominates, the threshold field should be independent of the size. At finite temperatures, however, super-paramagnetism reduces the threshold field, and thus the threshold increases with the size. We confirmed that the threshold field saturates when the grain size becomes larger than 20 nm, as depicted by open blue circles in Figure 18(a) where the effect of super-paramagnetism stops.

The threshold fields with and without DDI are depicted in Figure 18(a) as coercivity. Here, we clearly find that DDI reduces coercivity. In Figure 18(b), this result is shown as a function of grain size, where the data obtained in the literatures [86–89] are plotted by black circles. We find that coercivity tends to decrease as the logarithm of the linear dimension of the grain. The data obtained in Figure 18(a) are also plotted in the figure. There we find that the maximum point of coercivity exists as a function of grain size at finite temperature due to super-paramagnetism.

### 5.2. Coercivity in systems with multiple magnetic domains

For larger grains, the ferromagnetic configuration is no longer stable and a multidomain magnetic structure appears. Even in such large systems, a metastable uniform ferromagnetic state may exist. We studied this problem by using a simplified model:

(41) $$H=-\sum_{i,j}J_{ij}s_{i}\cdot s_{j}-\sum_{i}Ks_{zi}^{2}+\sum_{i,j}V_{DDI}(s_{i},s_{j}).$$

**Figure 19.**
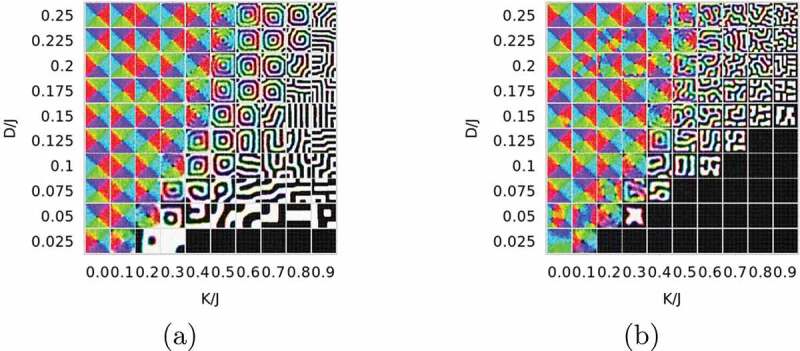
(a) Magnetic structures obtained by the thermal-quench process (A) for various values of anisotropies and DDI for systems of 64×64×10 at $T=0.3T_{C}$ where $T_{C}$ is the critical temperature of the bulk system. Out-of-plane component (top panels) and the in-plane horizontal component (bottom panel) are exhibited using the color code given in Figure 20. (b) Magnetic structures obtained by the field-quench process (B) from the out-of-plane ferromagnetic state

To investigate the parameter dependence of characteristic magnetic configurations, we surveyed the magnetic profiles under open boundary conditions with different anisotropy constants K, DDIs D, and thicknesses of systems $L_{z}$. We present magnetic profiles in a phase diagram in the space (K/J,D/J) for various thicknesses [44]. Five typical magnetic phases are found: out-of-plane ferromagnet, in-plane ferromagnet, vortex, multidomain, and canted multidomain. We depict examples for $L_{z}=10$ in Figure 19.

We evaluated coercivity by comparing the magnetic profiles obtained by the following two approaches:

(A) thermal-quench process in which the simulation starts from a random spin configuration at a high temperature, and then a MC update is performed at a given temperature to find a stationary state, which corresponds to the thermal demagnetization process, and

(B) field-quench process in which we switch off the magnetic field and observe the evolution of the system from a saturated ferromagnetic state obtained at a high field, which corresponds to the remanence process.

**Figure 20.**
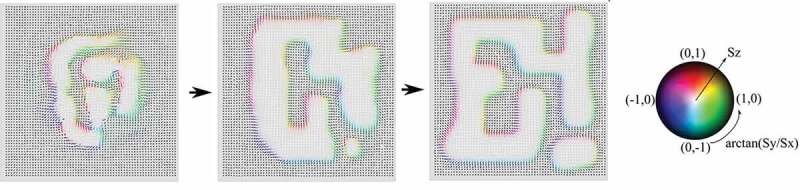
Nucleation pattern just after the collapse of out-of-plane ferromagnetic state. The spin direction $\left(S_{x},S_{y},S_{z}\right)$ is coded in the manner that $\left(S_{x},S_{y}\right)$ is given by color, e.g. (0.1) is red, and $S_{z}$ is coded by brightness of the color, i.e. the radius in the color code denotes $S_{z}$ from −1 (center) to +1 (edge). (From reference [44]: modified.)

Figure 19 indicates that the ferromagnetic state remains metastable in some parameter region where states obtained by the thermal-quench process are multidomain. This mechanism may give coercivity in rather large grains. The metastable ferromagnetic state collapses when D reaches a threshold. In Figure 20, we depict a configuration immediately after the collapse, where the magnetization reversal begins inside the plane. This nucleation is in significant contrast to the case of a nanoscale system, where the nucleation begins from corners [38,40].

## 6. Effect of grain boundary

The Nd${\;}_{2}$Fe${\;}_{14}$B magnet consists of hard-magnet (Nd${\;}_{2}$Fe${\;}_{14}$B) grains, each of which is covered by a grain boundary material [24,51,54,90]. Thus, it is important to study how boundary phases affect the coercivity of the grains studied in the previous section.

When we investigate nanocomposite magnets or magnets with soft-magnet defects, we consider α-iron as the boundary material. On the other hand, for sintered or hot-deformed magnets, the boundary phase consists of a Nd-rich material that shows a weak ferromagnetic property. The components of the soft phase have been studied by the concentration depth profile method [18], in which the width of the grain boundary is a few nanometers, and in most cases, it consists of a ferromagnetic soft material [15–19]. In the following, we study the former case in Subsection 6.1, in which the soft magnet first reverses its magnetization and reduces the coercivity of the hard magnet, which is called the spring effect. Then, we study nucleation and depinning phenomena in a sandwich structure of hard-soft-hard parts in Subsection 6.2, mainly focusing on the latter case. As a related problem, we study the effects of the surface in Subsection 6.3.

### 6.1. Effect of soft-magnet grain boundary on coercivity

**Figure 21.**
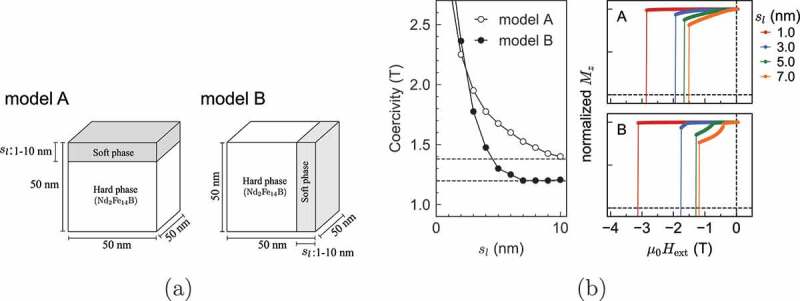
(a) Models with open boundary conditions in which the soft magnetic phase is placed on (001) surface (model A), and on (100) surface (model B), of the hard magnetic phase. (b) (left) Coercivities without DDI of models A and B as a function of soft phase thickness, $s_{l}$. (right) Parts of hysteresis loops for the models A and B with four different thicknesses $s_{l}$. (From reference [24]: modified)

Here, we study the spring effect due to a soft material. Because the exchange stiffness constant depends on the direction, the spring effect also depends on the direction. Thus, we studied magnetization reversal in systems with a soft phase in both directions [24]. We carried out micromagnetic simulations for two-phase models with the parameters, A(T) and K(T) for a finite temperature (T=400K), which were previously obtained in Section 3.3. The models are composed of soft and the hard mage exchange interactions among theses, respectively depicted in Figure 21(a), where the shaded parts denote the soft phases. Models A and B are the same if we do not take into account the anisotropy of A and DDI.

In Figure 21(b) (left), the dependence of coercivity on the thickness $s_{l}$ of the soft phase is depicted. Dashed lines denote the analytical results of the depinning-type coercivity [45,46]. In Figure 21(b) (right), the field dependences of the magnetization are plotted, in which changes in magnetization due to the nucleation in the soft layer and the depinning of the domain wall (i.e. the reversal of the hard magnet) are clearly seen.

Westmoreland, et al. [90] studied the effect of the soft magnet α-Fe by MC simulation and the SLLG equation using an atomistic Hamiltonian with thermal fluctuation. They used a simplified Hamiltonian that gives critical temperatures correctly, although the spin-reorientation transition is not realized. Using the model, they systematically studied the properties of core/shell nanocomposites with improved performance at a temperature suitable for motor applications (around 450 K).

### 6.2. Sandwich structure

To realize stronger coercivities at higher temperatures, it is necessary to study the effect of grain boundaries on the coercivity. For this purpose, a prototype hard-soft-hard magnet model, in which outer hard magnets are in contact with a middle soft magnet, has been intensively studied [45–50,91,92]. This model captures the essence of nucleation and depinning in inhomogeneous systems, and has been frequently used in analyses of the phenomena in various experimental and theoretical studies of magnetic materials [42,93] including GMR sensors [94].

Sakuma et al. investigated the threshold fields for nucleation and depinning in a hard-soft-hard magnet continuum model at zero temperature [45,48]. Solving a one-dimensional nonlinear equation for the model with the exchange stiffness constant and magnetocrystalline anisotropy energy, they presented a phase diagram of the threshold fields as a function of the ratios between the stiffness and anisotropy constants of the soft and hard magnets. However, thermal fluctuation effects, which are also essential for coercivity, were not studied.

Mohakud et al. [49] studied the temperature dependence of the corresponding phase diagram for the hard-soft-hard magnet model in the simple cubic lattice of the Heisenberg model with single-ion anisotropy by solving the SLLG equation (21,22). They showed various parameter dependences at different temperatures, and the threshold fields were found to be significantly affected by the thermal effect. Westmoreland et al. [50] studied this problem using atomistic and continuous spin models with temperature-dependent parameters for a (Nd magnet)-(α Fe)-(Nd magnet) system at finite temperatures and found that the thermal fluctuation reduced coercivity.

**Figure 22.**
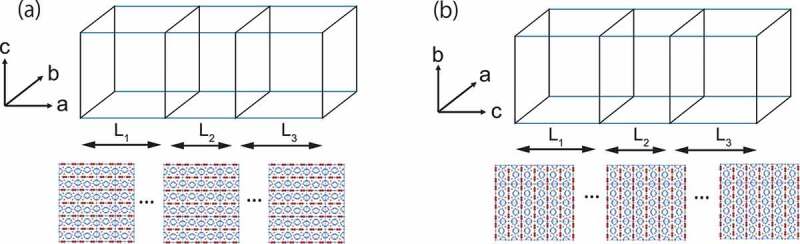
Systems of two bulk hard magnets (regions I(left) and III(right)) and a boundary soft magnet (region II(middle)). (a) system A, in which a domain wall runs along the a-axis (Bloch wall), (b) system B, in which a domain wall runs along the c-axis (Néel wall). The lower crystal structure is a view from the b- (a-) axis for system A (B). (From reference [51]: modified.)

**Figure 23.**
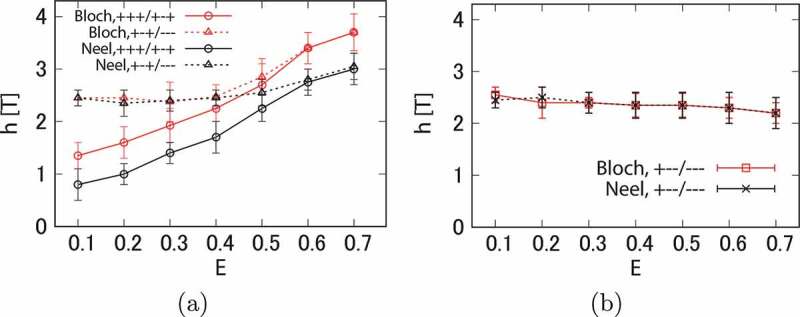
(a) Threshold fields for nucleation from (+++) to (+−+) (circles) and from (+−+) to (−−−) (triangles) for Bloch and Néel domain walls at 300 K. (b) Threshold field for depinning from (+−−) to (−−−) for Bloch (squares) and Néel (crosses) domain walls. Here, the temperature is 300 K, and $L_{1}=L_{2}=L_{3}=12$ (unit cells), and the ratio of exchange interactions of the soft and hard magnetic phases is 0.5 (F=0.5). Parameter E is the ratio of the anisotropy energies of the soft and hard magnetic phases multiplied by F. (From reference [51]: modified.)

These properties were studied using the atomistic model for the Nd${\;}_{2}$Fe${\;}_{14}$B magnet [51]. We do not have precise information on the detailed structure of the soft region, and we adopted the same lattice structure with reduced coupling constants and anisotropy energies. At about half of the critical temperature, the grain boundary is close to being paramagnetic, e.g. $T_{C}$ has been found to be about 200${\;}^{\circ }$C by X-ray magnetic circular dichroism (XMCD) [95]. Here, the details of the structure are not very relevant, and how coercivity of the hard magnet is reduced by the reversed magnetization in the grain boundary phase is an important issue. We depict the sandwich structure along the a- and c-axes in Figure 22. We define F and E/F, respectively, as the ratio of exchange interactions and that of anisotropy energies between the soft- and hard-magnet phases. In Figure 23(a), we plot for F=0.5 the threshold fields of nucleation, namely, for the process (+++) to (+−+) (circles) and also for the process (+−+) to (−−−)(triangles), in both cases. In Figure 23(b), the threshold fields of depinning, namely, for the process (+−−) to (−−−), are plotted.

It is found that the thermal fluctuation effects are considerably large in the Nd magnet, and at 300 K, the threshold fields of nucleation and depinning are much reduced and the E dependences are changed from those at T=0K [51]. Figure 23(a) and (b) show that the dependence of the threshold fields on the direction (Néel or Bloch type) is clear in the nucleation case, while the thresholds for depinning do not depend on the type. The threshold fields for the process for the process (+++) to (+−+) of the Bloch type are larger than those of the Néel type, which should be attributed to the stronger effective exchange interaction. The threshold fields for the process (+−+) to (−−−) are almost the same and constant for E<0.4, where the depinning of the reversed magnetization in region II gives the threshold. The threshold for the depinning process (right) is also almost the same and constant. In these cases, surface nucleation in region I or III is important for the depinning process, in which the strength of the magnetic anisotropy in region II is not essential, and the necessary field for nucleation is almost the same for the creation of Bloch and Néel domain walls.

### 6.3. Effect of surface properties

**Figure 24.**
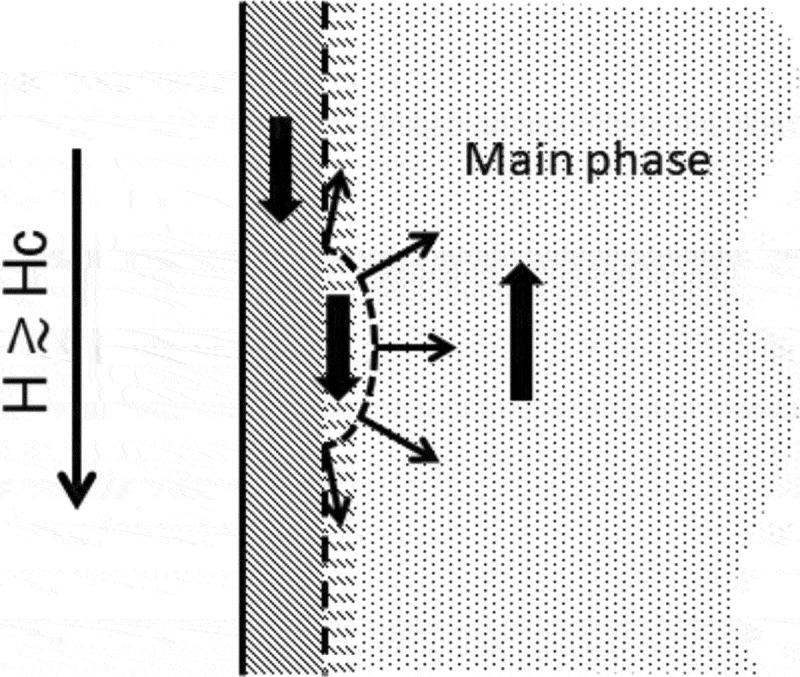
Domain wall depinning as a surface nucleation

As studied in the previous subsection, domain wall depinning is a process in which the reversed magnetization invades the neighbor hard grain (Figure 24). In this process, surface nucleation occurs under the influence of the contacting soft phase and the external field. The latter acts on the entire hard grain, while the former acts at the surface. Thus, the properties at the surface have significant effects on the coercivity of the magnet [96].

In this subsection, we study how the modification of the surface affects coercivity. For this study, we also used a system consisting of 12×12×9 unit cells. We studied the effects of modification of both the c-plane and the a-plane [52]. Here, we show the case of c-plane modification, where open and periodic boundary conditions are used along the c-axis and the a- and b-axes for the (001) surface, respectively.

It has been pointed out that at the surface, the anisotropy of a Nd atom is of the easy-plane type [97–99], in contrast to the easy-axis type in the bulk. On the other hand, surface easy-axis anisotropy may be enhanced by the substitution of strongly anisotropic atoms, for example, Dy [7,100]. Thus, we studied the three generic cases: (1) the anisotropy of modified layers is zero, (2) the anisotropy of modified layers is of the easy-plane type, and (3) the anisotropy of modified layers is of the enhanced easy-axis type. Concretely, we investigated coercivity with the following three settings of the anisotropy parameters $\left({\tilde{A}}_{2}^{0},{\tilde{A}}_{4}^{0},{\tilde{A}}_{6}^{0}\right)$ for the surface Nd atoms: (1) no anisotropy in Nd atoms: ${\tilde{A}}_{2}^{0}={\tilde{A}}_{4}^{0}={\tilde{A}}_{6}^{0}=0,$ (2) in-plane anisotropy in Nd atoms: ${\tilde{A}}_{2}^{0}=-A_{2}^{0}<0$ and ${\tilde{A}}_{4}^{0}={\tilde{A}}_{6}^{0}=0,$ where ${\tilde{A}}_{0}^{2}$ is negative and the amplitude is of the same order as that in the bulk [97–99], and (3) doubly reinforced anisotropy in Nd atoms: ${\tilde{A}}_{2}^{0}=2A_{2}^{0},{\tilde{A}}_{4}^{0}=2A_{4}^{0}$, and ${\tilde{A}}_{6}^{0}=2A_{6}^{0}$.

**Figure 25.**
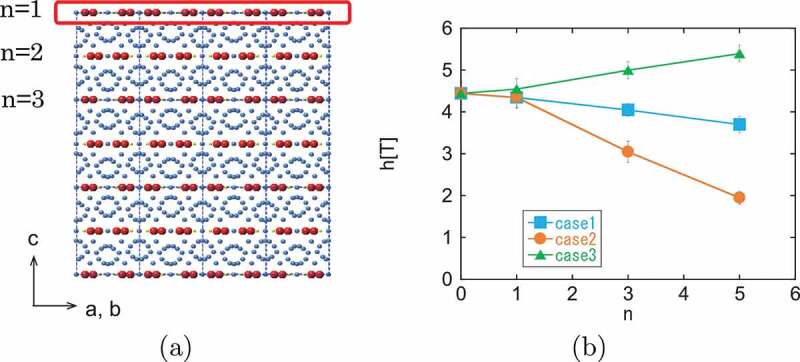
(a) Surface modification of the c-plane with n layers. (b) n dependencies of the threshold fields in cases (1)–(3) of surface modification (see the text) for the (001) surface at $T=0.46T_{c}$. (From reference [52]: modified.)

The dependence on the number of modified layers (n) is also important. In Figure 25(a), the definition of n is depicted. In Figure 25(b), the threshold fields for the magnetization reversal at $T=0.46T_{C}$ in the three cases are plotted as a function of n. At this temperature, the threshold field is much reduced from the value at T=0 [52,96], and also we find that single-layer modification (n=1) has little effect due to thermal fluctuation. However, as n increases, the effect becomes relevant. We suppose that if the width of the modified layer is comparable to the size of the activation volume, i.e. about half of the domain-wall width (n∼3), the modification becomes relevant. Thus, surface coating would be a useful method to increase coercivity.

In general, the properties of the surface of a grain are very important factors determining coercivity, and it has been reported that coercivity depends on the procedures on the surface of grains, for example, sputtering, heat treatment and grain boundary diffusion [100–102]. Thus, not only the modification of anisotropy, but also the surface roughness should be studied, which will be reported elsewhere [103].

## 7. Coercivity of magnets as an ensemble of grains

Magnets consist of grains, and the coercivity of a magnet must be studied by taking into account this feature. For the cooperativity of a magnet, the distribution of properties of grains (alignment, size, and shape) and also the interactions among grains including DDI play important roles.

As studied in previous sections, each grain has coercivity, which may be modeled by the threshold fields $H_{1}$ from up to down and $H_{2}$ from down to up. Such a unit representing a modeled grain is called a hysteron. Let us consider a magnetization process starting from a saturated state (all the hysterons are up) at a large positive field and reduce the field. In the process, each hysteron is reversed at its $H_{1}$, and the total magnetization changes with the field. If, at a field $H_{r}$, the field stops decreasing, and then increases, reversed hysterons remain in the down state until the field reaches $H_{2}$ of the grains. Thus, the total magnetization in the reverse process from $H_{r}$ $M(H_{r},H)$ shows a hysteresis that depends on $H_{r}$, which is called first-order reversal curve (FORC).

If hysterons are independent, the ensemble of hysterons is called the Preisach model [55]. There, the joint distribution of $H_{1}$ and $H_{2}$, $P(H_{1},H_{2})$, is obtained from $M(H_{r},H)$ by the following relation:

(42) $$P(H_{1}=H_{r},H_{2})={\left.\frac{\partial^{2}M(H_{r},H)}{\partial H_{r}\partial H}\right|}_{H=H_{2}}.$$

In real magnets, grains interact, and thus we cannot use the relation. However, the distribution obtained by the above relation is called a FORC diagram, which is often used to characterize the features of magnets [56–58]. It is an interesting problem to study how the interaction among the grains affects the distribution. The effect of the interaction has been taken into account in a mean-field-type analysis using the so-called moving (Preisach) model [59]. We are studying this dependence with an extended Preisach model (interacting Preisach model), in which the distributions of alignment, size, and shape are taken into account, which will be reported in the future [60].

## 8. Summary and discussion

We have reviewed works on finite-temperature properties by using the atomistic model. We first constructed an atomistic Hamiltonian describing the microscopic nature of the present material (2), where the intra-atomic electronic structure of the Nd atom (Figure 2) and the exchange interactions among the atoms were concretely set up. Then, the temperature dependences of various equilibrium properties were obtained. Then, the coercivity of nanograins was studied by the stochastic LLG method (Figure 10) and also by the free-energy landscape method using the Wang-Laudau MC algorithm (Figure 13). The temperature dependence of coercivity was also estimated quantitatively (Figure 15). A large reduction in coercivity was found, which gives the upper limit of coercivity at a given temperature. The magnetization reversal of individual grains has been experimentally observed [104] and the effect of thermal fluctuation on the process has become a realistic problem. The effect of DDI was studied by a newly developed SCO method, and we also studied the mechanism of coercivity in large systems with magnetic multidomain structures in Section 5.2. In this manner, we have studied coercivity from the microscopic scale to nanoscale.

The final goal should be the study of coercivity at the macroscopic scale. For magnets, ‘macroscopic’ does not simply mean a large size. Indeed, the simple enlargement of a system induces the multidomain state as mentioned above. Macroscopic magnets consist of an ensemble of grains. To study coercivity in the ensemble, we must take into account the cooperative behavior of grains. Thus, we studied the effects of grain boundary phases consisting of soft magnets in Section 6.1, and we also studied the temperature dependence of threshold fields for nucleation and depinning in the sandwich (hard-soft-hard) structure in Section 6.2. Moreover, in Section 6.3, we studied the effect of surface modification by changing the anisotropy energies of a few layers at the surface. There we found that the modification causes significant effects on coercivity. With this information on interactions among grains, in Section 7, we discussed possible extensions of the Preisach model [59,60] to study the effects of interactions on the FORC diagram which provides the characteristics of magnets [56,57].

### 8.1. Outlook for coercivity at several scales

Let us summarize the works from the so-called multiscale viewpoint. As shown in the present review, there are various scales at which coercivity can be studied. The main physical origin of coercivity is the anisotropy energy and the exchange energy, which is at the electronic scale (∼1Å). These quantities are studied by first-principles calculations, and reviewed for the present model in Section 2. The next scale is that of a single grain (∼ 20 nm). The coercivity of single small grains at finite temperatures was quantitatively estimated for the first time in the works presented in this review. It was found that the reduction due to the thermal fluctuation is rather large even in such simple small systems. In larger grains, DDI makes a uniformly magnetized state unstable and generates a magnetic multidomain (maze) structure. Indeed, magnetic domains have been observed in grains with a size of more than 100 nm, which is even smaller than the size of grains in sintered magnets (typically micrometers). However, the sintered magnets still show coercivity. We reviewed a work on coercivity in such cases. This problem must be studied more carefully in the future. Finally, we must consider coercivity at the macroscopic scale, that is, magnets that are an assembly of grains. At this scale, the cooperative motion of grains due to DDI and also the interaction with the neighboring sites play important roles in coercivity. The former effect has been studied as a demagnetization effect, but the effect may not be so simple because of the peculiar form of DDI. Namely, depending on the relative positions of spins, DDI may generate negative and positive fields. The effects of the latter have been studied in relation to domain wall depinning, and some attempts to study this phenomenon are reviewed in section 6. Studying all the effects microscopically is difficult, although several attempts have been made [53,54,105,106]. Thus, the combination of studies on the effects of boundary phases including atomistic first-principles approaches [107,108] and studies on ensemble effects such as the interacting Preisach model should be accelerated to understand the coercivity of magnets.

Finally, we mention the numerical calculations. To properly study the thermal effects of the present material, we used the SLLG method, the free-energy-landscape MC method, and a modified SCO method on the atomistic model. At the present computational capacity, we can use the atomistic model up to a size of 50 nm, which may include a single grain or a few grains. However, for further studies, we need to introduce a coarse-grained model. The relation between the atomistic model of the present work and the continuum magnetization model used in the so-called micromagnetic simulation (LLG equation) is an important problem. We constructed a continuum magnetization model by obtaining the stiffness constant and the anisotropy energy at a given temperature as reviewed in Section 3.3 [24]. However, to directly perform a simulation with the thermal fluctuation, it is necessary to introduce a model consisting of local variables. A renormalization analysis has been attempted by introducing coarse-grained magnetization with a variable length, where magnetization distribution was obtained by the Wang–Landau method. For a unit cell, the magnetization distribution P(M) is given by

(43) $$P(M)\propto e^{-\beta E_{self}(M)}=\int_{microscopicconfigurations}e^{-\beta H}\times \delta \left(M\;-\sum_{i}S_{i}\right).$$

Using this variable-length magnetization, the Hamiltonian of the system may be approximated by

(44) $$H_{\;\;\;M\;\;}=\sum_{ij}J_{ij}\;M_{i}\;\;\cdot \;\;\;M_{j}+\sum_{i}E_{self}(M_{i}).$$

**Figure 26.**
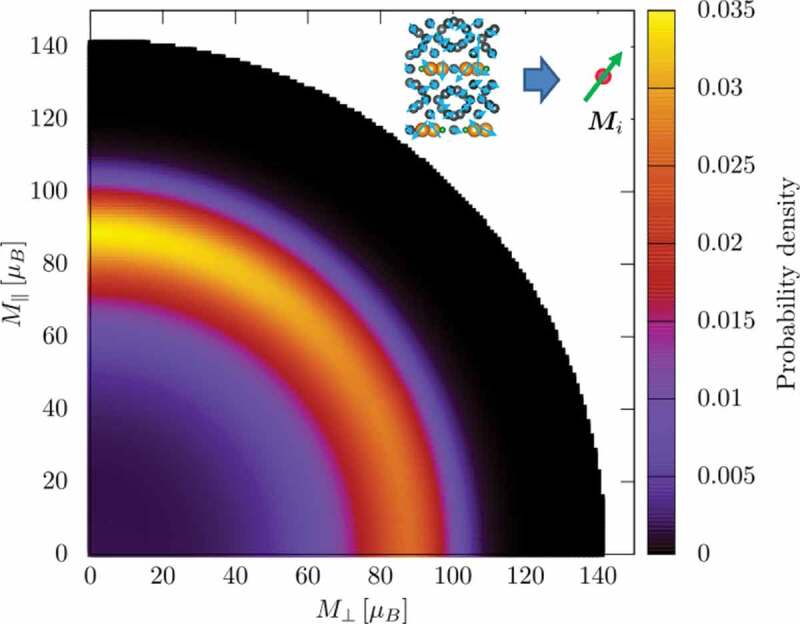
(a) Effective potential energy as a function of magnetization $\left(M_{⊥},M_{∥}\right)$ of a unit cell at T=400K. Distribution of magnetization is peaked at around 90$\mu_{B}$ on the c-axis $\left(M_{∥}\right)$

As we see in Figure 26, the magnetization of an isolated unit cell is about 90$\mu_{B}$. However, in a crystal consisting of unit cells combined by ferromagnetic interactions, it was found that the average length of magnetization is increased by the interactions among them to about 116$\mu_{B}$, which agrees with the experimental value. To refine the parameters used to reproduce atomistically obtained properties, the tuning of renormalized parameters is necessary, which depends on the quantity on which we are focusing; details are under investigation.

For real-time dynamics, the conventional MC method using the detailed balance is not adequate and LLG-type real-time dynamics is necessary. We have introduced the SCO algorithm to accelerate the calculation of systems with DDI. However, the applicability of the SCO method, which is based on the time evolution given by the detailed balance, to the LLG equation is not known. We have developed the so-called time-quantified MC method in which real dynamics such as the precession of spins are correctly simulated [109]. The developments of these techniques are also desirable for further studies.

## Appendix A: exchange constants and magnetic moments of Nd2Fe14B used in the present work

We used the exchange parameters obtained by Akai-KKR (MACHIKANEYAMA) [110], which produces the exchange coupling constants with the Liechtenstein method [65], using the experimental lattice parameters at room temperature in Ref [2]. The list of exchange coupling constants is given in Supplementary material 1. In the list, in ‘Atom positions’, the first column is the sequential number of atoms in a unit cell and the following three columns give the position of the atom. The last two columns give the type and spin moment ($\mu_{B}$) of each atom. In ‘Exchange data’, exchange constants including both spin amplitudes are given in the following manner: The first column (bond) is the sequential number of bonds. The second and third columns are the atom numbers (atom1, atom2) connected by the bond. The next three columns (cell) denote the position of the unit cell in which atom2 exists. For example, (0.0000 0.0000 − 1.000) means that atom2 is in the unit cell below the main unit cell in which atom1 exists. The last two columns give the distance between the atoms and the exchange coupling in unit of meV.
